# Influence of Stress and Depression on the Immune System in Patients Evaluated in an Anti-aging Unit

**DOI:** 10.3389/fpsyg.2020.01844

**Published:** 2020-08-04

**Authors:** Beatriz Cañas-González, Alonso Fernández-Nistal, Juan M. Ramírez, Vicente Martínez-Fernández

**Affiliations:** ^1^Department of Psychobiology, Facultad de Psicología, Universidad Nacional de Educación a Distancia (UNED), Madrid, Spain; ^2^Department of Pharmacology and Toxicology, Facultad de Farmacia y Nutrición, University of Navarra, Pamplona, Spain; ^3^Department of Morphological Sciences, School of Medicine, University of Córdoba, Córdoba, Spain

**Keywords:** stress, depression, biological markers, exercise, cognition

## Abstract

**Background:**

There is compelling evidence pointing out that stress and depression produce a dramatic impact on human well-being mainly through impairing the regular function of the immune system and producing a low-chronic inflammation status that favors the occurrence of infections, metabolic diseases, and even cancer. The present work aims to evaluate the stress/depression levels of some patients treated in an antiaging unit and detect any potential relationship with their immune system status prior of the implementation of a physical/psychological program designed to prevent health deterioration.

**Methods:**

We evaluated 48 patients (16 men and 32 women with a mean age of 55.11 ± 10.71 years) from middle-upper class from psychological and immunological points of view. In particular, we analyzed neutrophil chemotaxis and phagocytosis; lymphocyte chemotaxis and proliferation, and natural killer (NK) cell activity.

**Results:**

Women showed more depressive symptoms than men. Chemotaxis levels of lymphocytes and neutrophils in women showed a significant reduction compared with those in men. We also found a strong negative correlation between depression and NK cell function. This correlation was also significant independently of gender.

**Conclusion:**

We conclude that NK activity is affected at least by depression state, and we propose that a combined treatment consisting of cognitive behavioral therapy and physical activity programs might improve patient health deterioration.

## Introduction

Psychoneuroimmunology (PNI) is a multidisciplinary science that is focused on the interaction between the brain and the immune system and the possible clinical outcomes (Solomon et al., 1997). Clinically, PNI includes the knowledge of biological mechanisms subordinated to underlying psychosocial events that start and/or develop the immunological disease and the understanding of the immunological responses generated in psychiatric diseases. In the last part of the 1950s and at the beginning the 1960s, Rasmussen et al. (1957) elegantly demonstrated in laboratory rats the connection between aversive learning and the susceptibility to the infection produced by herpes virus. Independently in the 1970s, Solomon and de Vessye reported the first correlation between suffering long stress periods and the decreased antibody reactivity in animal models (Solomon, 1969; Solomon et al., 1974). Thus, PNI might represent a link among different disciplines such us psychiatry, psychology, neurology, endocrinology, immunology, neurosciences, internal medicine, and even surgery (wound healing).

Evidence supports the interaction between neuronal and immune systems. Thus, preliminary studies reported that psychosomatic observations were related to some autoimmune disease such as lupus erythematosus, Grave’s disease, and rheumatoid arthritis (Solomon, 1969). Then, in the 1980s, it was demonstrated that lymphocytes were able to synthesize adeno corticosterone and β-endorphin molecules and not only produced exclusively by neuronal cells (Blalock et al., 1985). Guillemin et al. (1985) reported that hypothalamic response is increased after antigen administration and that the hypothalamic–pituitary–adrenal (HPA) axis is also activated by antigen presence and pro-inflammatory cytokines resembling stress conditions. The influence of stress, anxiety, and depression in allergies, dermatitis, and asthma has also been reported. These studies point to a delayed response of T and B cells which are activated in these situations (Gil et al., 1987; Pariante et al., 1994; Djurić et al., 1995). There is ample evidence supporting that depression and stress are related to cancer development (Burnet, 1971; Nakaya, 2014). Thus, in stress animal model, it has been reported a diminution of lymphocyte proliferation, natural killer (NK) cell activity, cytokine production, and an increase in the tumor size, angiogenic process, and metastasis (Monjan and Collector, 1977; Visintainer et al., 1982; Glaser et al., 1985; Ben-Eliyahu et al., 1991; Wu et al., 2000). Nowadays, there are compelling evidences demonstrating that pro-inflammatory status influences neurological function leading to changes in serotonin production which in turn induces depression (Leonard, 2010).

In humans, several studies have reported that depression or stress situations such as bereavement, a divorce, demanding work environment, or students in exam periods produce a decrease in NK, neutrophil, and lymphocyte activities such as lysis, proliferation, and chemotaxis, making the activities of these immune cells good markers of immune status in patients (Irwin et al., 1988; Schleifer et al., 1996; Andersen et al., 1998; Benschop et al., 1998; Byrnes et al., 1998; Bosch et al., 2005; Arranz et al., 2009; Boscolo, 2009). Stress and depression also modulate the production of hormones such as adrenaline, corticoids, and catecholamine that in turn influence the immune system (Leonard, 2010). It has been reported that stress, depression, and inflammation can activate and modify cytokine homeostasis (Kiecolt-Glaser et al., 2003). Cytokines might have a depressive effect well directly through releasing corticotrophin or indirectly increasing the resistance to activation of glucocorticoid receptors. This will cause a system hyper-activation due to the suppression of the normal feedback mechanism of the HPA axis.

Lymphocytes, neutrophils, and NK cells play an important role in the immune response against pathogens and tumor cells. Currently, it is easy to evaluate in a sample of blood oxidative activity and phagocytosis of neutrophils and its chemotaxis, which is to quantify the movement of the mentioned cells in a gradient of chemotactic agents (Matzner, 1987; Lord, 1989). In particular, Lymphocyte Chemotaxis Index (LCI), Neutrophil Chemotaxis Index (NCI), Neutrophil Phagocytic Index (NPI), lymphocyte proliferation (LP), and NK activity (NK lysis %) are markers routinely used for immune function evaluation. Immune biomarkers have been found altered in relation to stress or depression in previous studies (Khanfer et al., 2010; Vitlic et al., 2014; Duggal et al., 2015, 2016). Thus, chronic stress reduces chemotaxis of peripheral blood of mononuclear cells (Merry et al., 1996; Redwine et al., 2004), and phagocytic activity of neutrophils (PAN) has been reported downregulated in clinically depressed patients (McAdams and Leonard, 1993) as well as LP and NK activity have also been reported decreased (Esterling et al., 1996; Andersen et al., 1998; Scanlan et al., 2001; Gan et al., 2002; McGuire et al., 2002).

Nowadays, and given the aging population, more people want to receive a specific treatment focused on alleviating or delaying the effects of aging to get a better quality of life. We also believe that a psychological/physical program designed to prevent physical and physiological deterioration might achieve this goal. However, before implantation of this program, it would be necessary to know the depression and stress levels of each patient and his or her immunological status. Thus, we aimed to characterize the immune status in an middle-upper class population that is often under stressful conditions. We also wanted to explore if the patient’s gender, given the differences between men and women from a psychological and physiological point of view, might affect these evaluations. Thus, in this study, we aimed to evaluate if there was any association between stress and depression status in patients who are under study in an antiaging unit and the levels of immune response markers in these patients prior to the implementation of a physical activity program to prevent physical and psychological deterioration.

## Materials and Methods

### Study Design and Psychological Evaluation

We analyzed the immune system status of 48 patients (32 women and 16 men) who are classified under middle-upper class population according to Spanish settings who developed their professional life under stressful conditions. The patients were evaluated in an antiaging unit following a psyche–clinical evaluation to determine its basal status regarding biological age from a physical and psychological point of view.

We evaluated psychologically the patients through Beck Depression Inventory (BDI) (Beck et al., 1988) and Perceived Stress Questionnaire (PSQ) (Sanz-Carrillo et al., 2002). Briefly, the BDI is a questionnaire that includes 21 items, each including four alternative statements ranging in order of severity from 0 to 3. Conventional cutoffs are 0–9 for normal range, 10–18 for mild to moderate depression, 19–29 for moderate to severe depression, and 30–63 for severe depression. Regarding the PSQ, this questionnaire evaluates with 30 items the stress levels of the patient. The questions have 4 degrees ranging from 1 equaling almost never to 4 meaning almost always. Then, an index is obtained that can range from 0 (low level of perceived stress) to 1 (high level of perceived stress).

This study was carried out following the guidelines of the Helsinki Declaration on human studies. The scientific-ethical board of the center approved this study. All patients were previously informed and signed the corresponding informed consent.

This study was carried out in our antiaging unit of our institution. Briefly, the antiaging unit focused on providing psychological and medical attention to patients who wanted to prevent their physical and cognitive deterioration. The patients were evaluated from a medical and psychological point of view, and the patients received a report in which the medical–psychological team decided the treatment that included pharmacological and psychological therapy together with physical exercise and nutritional recommendations.

### Blood Sample Collection

Patients’ blood samples were taken by venipuncture from an inner fold arm vein with Vacutainer^®^ tubes between 8 and 10 a.m. and under starvation conditions. For serum hormone levels, one tube of 5 ml (containing separating gel and clot activator) was collected per patient. For immune cell functional assays, including lymphocyte proliferation, neutrophil phagocytic, lymphocyte/neutrophil migration, and NK activity assays, two tubes of 10 ml (sodium heparin) were collected per patient.

We evaluated the hormonal levels in the patients’ blood samples, sexual hormones (progesterone, testosterone, estradiol 17β, prolactin), thyroids hormones [thyroid-stimulating hormone (TSH) and thyroxine (T4)], insulin, and insulin-like growth factor I (IGF-I).

### Immune System Status

Specifically, regarding the immune system, we performed neutrophil function analysis chemotaxis index (NCI) and phagocytosis index (NPI); lymphocyte function analyses, chemotaxis index (LCI), and proliferation (LP); and NK activity (lysis %).

#### Neutrophil and Lymphocyte Isolation, Lymphocyte Proliferation Assay

Neutrophils were separated from peripheral blood using a Miltenyi magnetic column. Briefly, blood from a heparinized tube was labeled with antibodies against lymphocytes, monocytes, and leukocytes [antibodies against CD2, CD5, CD45R, and F4/80 and intercellular adhesion molecule (ICAM)-I]. Blood was added to a MACS column, and then passed through fraction was collected as neutrophil fraction (Cotter et al., 2001).

#### Lymphocyte Isolation and Proliferation Assay

We isolated the lymphocytes from Ficoll-Hypaque peripheral blood mononuclear cells of heparinized samples of patient’s blood. After that, we adjusted lymphocyte concentration to 5 × 10^6^ cells/ml and added mitogen stimulus phytohemaglutinin (PHA); no PHA was added to controls. We culture the cells for 68 h and then incubate the cells with 0.5 mCi of ^3^H-thymidine for 4–6 h. We filtered the cultures with Whatman paper, and the filters were dried and counted in a γ-counter. Proliferation index was calculated as (cpm PHA stimulated cultures)/(cpm non-PHA stimulated cultures).

#### Neutrophil Phagocytic Assay

We evaluated PAN using Phagotest kits (Orpegen Pharma GmbH, Heidelberg, Germany) within 2 h of blood extraction. This kit evaluates neutrophil phagocytosis of fluorescein isothiocyanate-labeled opsonized *Escherichia coli*. Whole blood (100 μl) was incubated with 20 μl of fluorescent bacteria (2 × 10^7^) at 37°C for 10 min, whereas a negative control sample remained on ice. Using a flow cytometer using a blue-green excitation light 488 nm argon-ion laser, we measure the mean fluorescent intensity corresponding to the number of bacteria phagocytosed by neutrophils.

#### Migration Assays for Neutrophil (Neutrophil Chemotaxis Index) and Lymphocytes (Lymphocyte Chemotaxis Index)

After isolation of mononuclear white cells from peripheral blood, we proceeded to migration assays. Chemotaxis assays were performed on Transwell chambers with 5.0 mm pore size inserts (Corning, Corning, NY, United States) as previously described (Figueroa-Vega et al., 2010). Lower chambers were filled up with RPMI-1640 culture media supplemented with 0.2% human serum albumin with or without recombinant human angiopoietin (Ang)-1 or Ang-2 at 100 ng/ml (R&D Systems). Monocytes (5 × 10^5^ in 100 ml) were seeded into the upper chamber. After 2 h of incubation at 37°C, filters were removed, fixed, and stained with 4′,6-diamidino-2-phenylindole. Cells that had migrated and were attached to the lower side of the membrane were counted per field with an epifluorescence microscope. Results were expressed as the average number of migrated cells.

#### Natural Killer Cell Activity (Lysis %)

We have assayed NK cell activity as previously described (Davis et al., 2011). First, we isolated peripheral mononuclear cell from the blood of the patient that we have previously collected in heparinized tubes. Then, the NK cells were isolated using an NK Cell Isolation Kit (Miltenyi Biotec) according to the manufacturer’s instructions. We stimulated NK activity by incubating the cells with interleukin (IL)-2 (100 U/ml), IL-18 (20 ng/ml), and IL-12 (5 ng/ml) overnight at 37°C. Then, we characterized NK cell cultured population using a fluorescence-activated cell sorting (FACS) analysis with a sample of cells that were added to 200 μl of PBE buffer: phosphate buffered saline (PBS), pH 7.2 0.5% bovine serum albumin (BSA), and 2 mM ethylenediaminetetraacetic acid (EDTA), with 5 μl of phycoerythrin (PE)-conjugated anti-CD56 and 5 μl PE/Cy5-conjugated anti-CD3 (Abcam). We labeled target cells K562 (5 × 10^5^ cells) with 100 μCi of ^51^Cr for 1 h at 37°C in 5% carbon dioxide (CO_2_). Then NK cells were added to K562 cells in an effector–target ratio of 1:1 and incubated for 4 h a 37°C in 5% CO_2_. After that, the supernatant was aspirated, and ^51^Cr was measured in a γ-counter. We calculate lysis % following the formula [experimental release – spontaneous release]/[maximum release – spontaneous release] × 100.

### Statistics

We use Mann–Whitney *U*-test to find out the differences in age between men and women. Spearman correlation (R) test was used to find the possible associations. SPSS v21 software ran the statistical tests. A *p* < 0.05 was considered significant.

## Results

### Patient Description

This study included 48 patients who were visiting our antiaging unit in 2017. The average age was 55.11 ± 10.71 years with a variation coefficient of 19%. Data by gender are presented in Table 1. There were no significant differences in age between men and women. The hormone levels were within normal ranges for their respective ages (Table 1).

**TABLE 1:** Patient’s data and hormonal values.

**Patients**	**Men**	**Women**	**Reference values**
Number	16 (%, 33.3)	32 (%, 66.7)	n.a.
Age	52.56 ± 11.11	55.41 ± 10.16	n.a.
**Sexual hormones**			
FSH (mUI/ml)	7.2 ± 3.9	84.6 ± 24.6	Men: 1.4–18.1 Women: 0.5–76*
LH (mUI/ml)	5.3 ± 2.5	41.3 ± 17.8	Men: 1.5–9.3 Women: 0.5–76*
Estradiol 17-β (pg/ml)	n.d.	107.6 ± 53.9	Women 11–196*
Progesterone (ng/ml)	n.d.	11.8 ± 4.9	Women: 1–20
Prolactin (ng/ml)	n.d	14.7 ± 7.0	Women: 1.8–29.2
Testosterone (ng/ml)	3.6 ± 2.0	n.d.	0.86–7.88
**Thyroid hormones**			
TSH (μUI/ml)	3.4 ± 1.4	2.9 ± 1.6	0.35–5.5
T4 (ng/dl)	1.4 ± 0.4	1.3 ± 0.5	0.78–1.8
Insulin (mg/dl)	84.5 ± 11.2	78 ± 11.6	60–100
IGF-I (ng/ml)	131.4 ± 55	111.2 ± 48.1	43–220

Data are mean ± standard deviation. n, number of patients. *Depends on menstrual cycle and postmenopausal status. n.a., not applicable; n.d., not determined. Estradiol 17β, progesterone, and prolactin levels were evaluated only in women, whereas testosterone was evaluated only in men. FSH, follicle-stimulating hormone; TSH, thyroid-stimulating hormone; LH, luteinizing hormone; IGF-I, insulin-like growth factor I.

### Depression and Stress Levels

**FIGURE 1:**
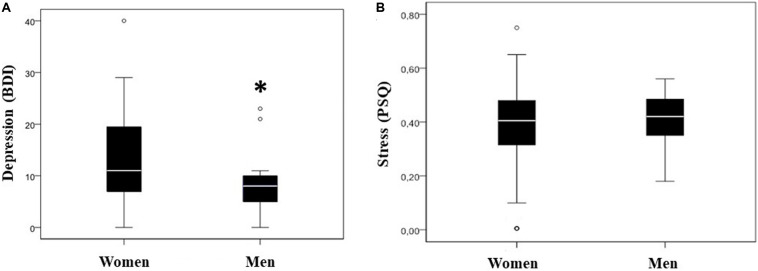
Stress and depression levels. Box and whisker plots of stress and depression levels of the population under study (median is represented with a white bar). Depression levels evaluated by Beck Depression Inventory (BDI) **(A)**. BDI levels 0–9 for normal range, 10–18 for mild to moderate depression, 19–29 for moderate to severe depression, and 30–63 for severe depression. ^∗^*p* < 0.05 vs. women depression levels. Stress levels evaluated by Perceived Stress Questionnaire (PSQ) **(B)**. PSQ level 0 means a low level of perceived stress, whereas a value of 1 is a high level of perceived stress.

One of our first tasks was to analyze depression and stress levels in our population. When we analyzed the levels of depression and stress in both men and women, we found that depression levels were significantly higher in women than in men (*p* < 0.05), mean difference 5.06; IC_95%_ = -0.062–10.19 (Table 2 and Figure 1A). Then, we wanted to find out the stress levels in our patients. In this case, we did not find any differences in the stress levels between both genders (Table 2 and Figure 1B).

**TABLE 2:** Depression and stress levels.

	**Men**	**Women**	***p***
Stress	0.41 ± 0.10	0.38 ± 0.18	0.501
Depression	8.75 ± 5.92	13.81 ± 9.25	**0.027**

*Data are mean ± standard deviation. *p* is the associated value to the corresponding statistical test.*

### Immune Status by Gender

**FIGURE 2:**
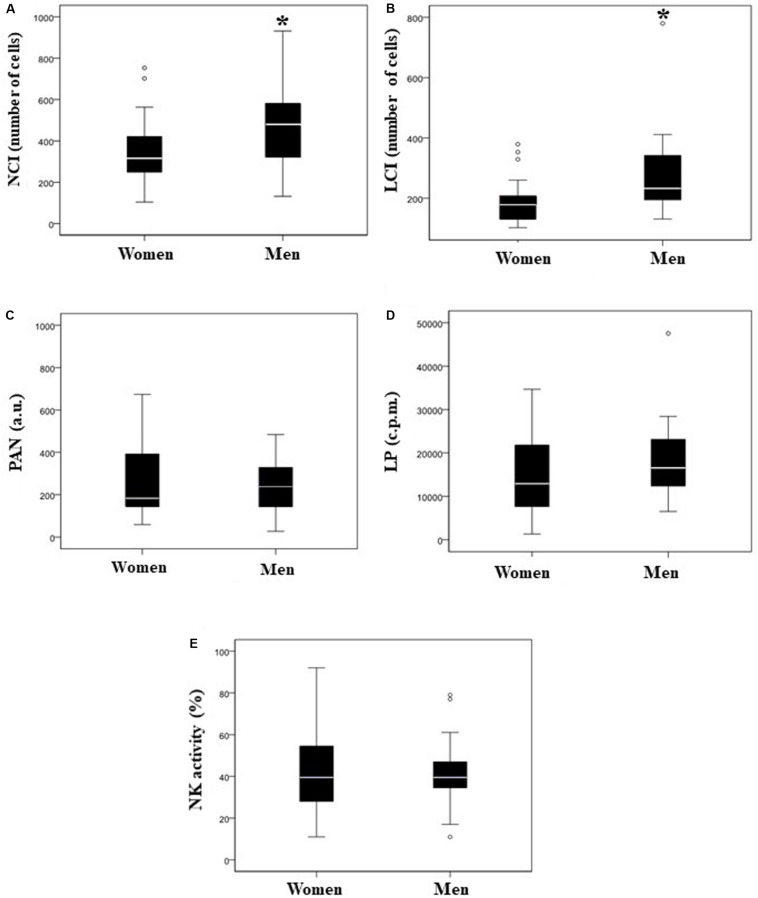
Comparison of immune response in both sexes; box and whisker plots. Neutrophil Chemotaxis Index (NCI; number of cells) **(A)**, Lymphocyte Chemotaxis Index (LCI; number of cells) **(B)**, phagocytic activity of neutrophils (PAN; a.u., arbitrary units) **(C)**, lymphocyte proliferation (LP; c.p.m.) **(D)**, and natural killer (NK) activity **(E)**. White bar denotes the median. *p < 0.05 vs. women.

Our next step was to figure out if there were differences in immune system response regarding gender. Thus, we found that men showed significantly (*p* < 0.05) higher values in the NCI and LCI values than women (Table 3 and Figures 2A,B). However, regarding PAN, LP, and NK activity, we did not find any significant differences (Table 3 and Figures 2C–E).

**TABLE 3:** Immune status by sex.

**Marker**	**Men**	**Women**	***p***
NCI	468 ± 29.27	350 ± 21.15	**0.032**
PAN (a.u.)	237 (144–329)	184 (147–393)	0.965
LCI	291 ± 22.46	185 ± 10.14	**0.002**
LP (cpm)	18,493 ± 1,481	15,202 ± 1,440	0.279
NK (lysis %)	42 ± 2.75	42 ± 7.24	0.955

*NCI, Neutrophil Chemotaxis Index (number of cells); PAN, phagocytic activity of neutrophils (a.u., arbitrary units; range in brackets minimum and maximum); LCI, Lymphocyte Chemotaxis Index (number of cells); LP, lymphocyte proliferation (cpm); NK, natural killer. Data are mean ± SEM. *p* is the associated value to the corresponding statistical test.*

### Correlations Between Stress, Depression, and the Immune System

**FIGURE 3:**
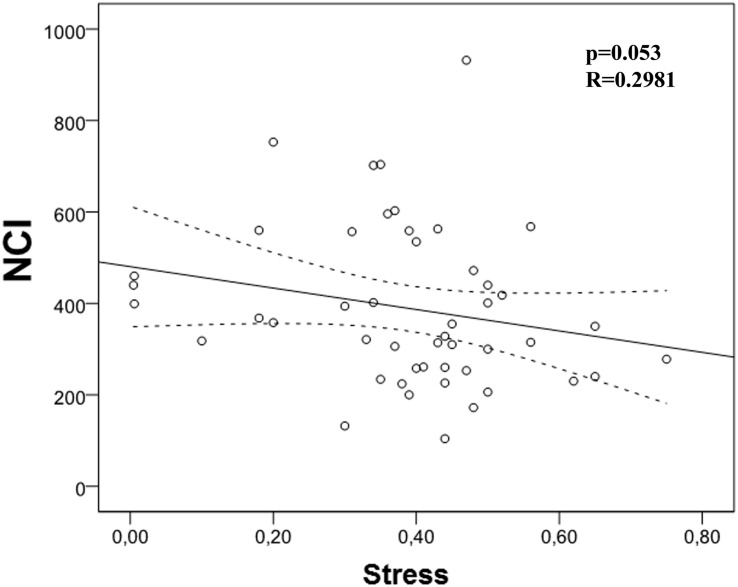
Correlation between Neutrophil Chemotaxis Index (NCI) and stress. There is a strong trend correlation between NCI and stress (*p* = 0.053, *r* = -0.281).

**FIGURE 4:**
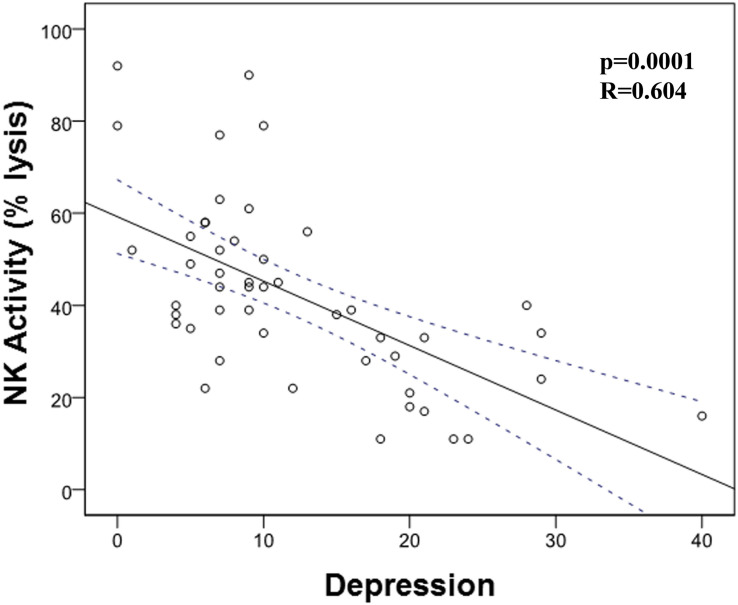
Correlation between natural killer (NK) cell activity and depression. There is a significant correlation between NK activity and depression (*p* = 0.001; *R* = -0.604).

Then, we analyzed the possible correlation between stress and the different immunological markers under evaluation. We found a trend (*p* = 0.053) between NCI and this stress (Table 4 and Figure 3). However, we did not find any correlation between PAN, LP, NK activity, and stress. When we carried out this correlation analysis with depression, we found a strong correlation between NK activity and depression (Table 5; *p* = 0.001; *R* = -0.604, Figure 4), pointing out that when depression level is higher, the NK activity decreases.

**TABLE 4:** Stress and immune marker correlations.

**Marker**	**R Spearman**	***p***
NCI	–0.281	0.053
PAN	–0.083	0.575
LCI	–0.062	0.677
LP (cpm)	–0.095	0.503
NK (lysis %)	–0.017	0.908

*NCI, Neutrophil Chemotaxis Index; PAN, phagocytic activity of neutrophils; LCI, Lymphocyte Chemotaxis Index; LP, lymphocyte proliferation; NK, natural killer.*

**TABLE 5:** Depression and immune marker correlations.

**Marker**	**R Spearman**	***p***
NCI	0.001	0.994
PAN	–0.116	0.433
LCI	–0.015	0.919
LP	–0.096	0.514
NK activity (lysis %)	–0.604	0.001

*NCI, Neutrophil Chemotaxis Index; PAN, phagocytic activity of neutrophils; LCI, Lymphocyte Chemotaxis Index; LP, lymphocyte proliferation; NK, natural killer.*

### Correlation of Immune Markers With Age

**FIGURE 5:**
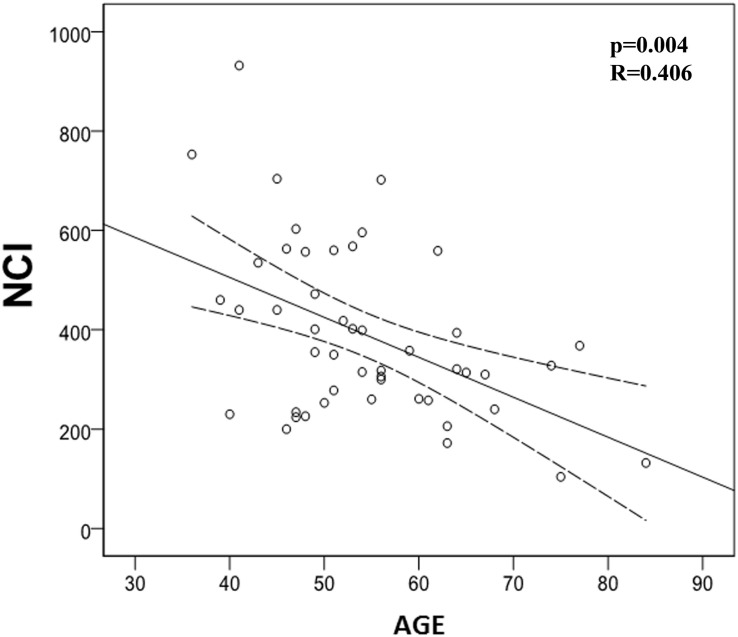
Correlation between Neutrophil Chemotaxis Index (NCI) and age. There is a significant negative correlation between NCI and age (*p* = 0.004; *R* = -0.406).

**FIGURE 6:**
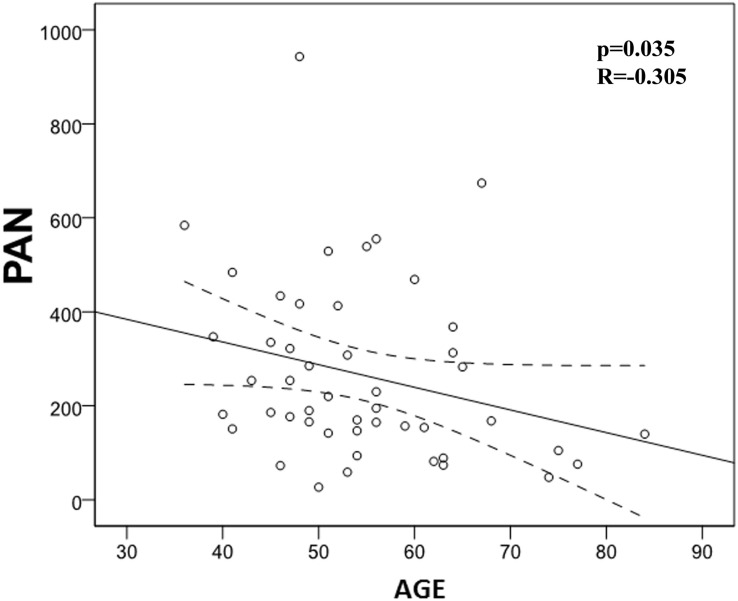
Correlation between phagocytic activity of neutrophils (PAN) and age (*p* = 0.035; *R* = -0.305). There is a significant negative correlation between PAN and age.

We also evaluated the possible correlation between quantified studied immune markers and patients’ age. In this regard, we found a significant negative correlation between age and NCI (*p* = 0.004) and PAN (*p* = 0.035), meaning that immune system activation is impaired with age (Table 6 and Figures 5, 6). However, we did not find any correlation between LCI, LP, or NK activity with age.

**TABLE 6:** Correlation between immune markers and age.

**Marker**	**R Spearman**	***p***
NCI	–0.406	**0.004**
PAN	–0.305	**0.035**
LCI	–0.239	0.101
LP	–0.204	0.165
NK activity (lysis %)	–0.016	0.913

*NCI, Neutrophil Chemotaxis Index; PAN, phagocytic activity of neutrophils; LCI, Lymphocyte Chemotaxis Index; LP, lymphocyte proliferation; NK, natural killer. *p* is the associated value to the corresponding statistical test.*

### Differences in Correlations by Gender

We analyzed separately the correlations by gender, and we found that the trend between stress and NCI was not significant for men but it was significant for women (Table 7). However, the correlation between depression and NK activity was also significant for each gender (Table 8).

**TABLE 7:** Correlation between stress and NCI in both sexes.

**Correlation**	**R Spearman**	***p***
NCI and men	–0.060	0.824
NCI and women	–0.372	**0.036**

*NCI, Neutrophil Chemotaxis Index. *p* is the associated value to the corresponding statistical test.*

**TABLE 8:** Correlation between stress and NK activity in both genders.

**Correlation**	**R Spearman**	***p***
NK activity (lysis %) and men	–0.625	0.010
NK activity (lysis %) and women	–0.648	0.001

*NK, natural killer.*

## Discussion

It is well accepted that a good psychological status favors a better immune response against disease (Solomon, 1969; Solomon et al., 1974; Connor and Leonard, 1998; Bauer et al., 2009; Dragoş and Tănăsescu, 2010; Leonard, 2010; Stojanovich, 2010; Robson et al., 2017). Thus, several reports demonstrate that improving the psychological status through the use of social support techniques is related to an increase in survival in women with metastatic breast cancer or melanoma (Spiegel et al., 1989; Fawzy et al., 1993; Mustafa et al., 2013; National Collaborating Centre for Cancer [UK], 2015). Also, neuropsychological interventions using physical exercise of different tools related to cognitive improvement appear to slow cognitive decline (Levin et al., 2017). However, the effectiveness of psychological interventions in improving patient’s biological status is under debate. Thus, a recent meta-analysis analyzing this relationship reported little evidence between the benefits of psychological treatments for depression and positive biological outcomes. This lack of effect might be due to methodological inconsistencies in the revised studies (Cristea et al., 2019).

Here, our aim was to characterize the status of immune response and its correlation with stress and depression in a group of patients before their participation in a psychological/physical program that designed to prevent physical and physiological deterioration. We treated more women (66.7%) than men (33.3%) in our antiaging unit in 2017. Of note, there were no significant differences in age between men (52.56 ± 11.11 years old) and women (55.41 ± 10.16 years old).

Women showed a basal level of depression of 13.81 in BDI related to a low-level depression. However, men only reach 8.75 points in this test, which is associated with no depression at all in BDI. It has been reported that women are twice likely to suffer depression during their life than men, and this might be caused by multiple reasons such as hormonal differences, cultural backgrounds, gender differences in social activities, and response to stressful situations (Wharton et al., 2012; Altemus et al., 2014; Kuehner, 2017). In our study, one of the causes of this ostensible increase might be explained because they are close to perimenopause period. Huge life changes in this period might be related to endocrine metabolic, sexual activity, and even family conflicts that lead to anxiety and depression (Gyllstrom et al., 2007; Maki et al., 2018; Willi and Ehlert, 2019). Regarding basal stress levels evaluated through the PSQ questionnaire, both genders showed medium scores that means that men and women are inside the range of moderate stress (men 0.41 and women 0.38), although there were no significant differences. This moderate stress levels might be related to a high working demanding environment (senior executives), aging related to a perception of cognitive and physical outputs, and even with the social commitments belonging to their high economic level.

In our study, we have found significant differences between genders in NCI and LCI parameters being both significantly greater in men than in women. We have to underline that our patients are stressed but not depressed or in the lower phase of depression and probably they are not yet immunologically affected, which makes them good candidates for a prevention program. In fact, our hypothesis was that we might find some signs of immune function deterioration in our patients. Indeed, we have found a negative correlation between depression and NK activity, meaning that higher depression level is associated with a lower cytotoxic NK activity, which concurs with previous reports (Zorrilla et al., 2001) where the authors reported in a meta-analysis review an overall leukocytosis, a reduced NK cell activity, and a poor proliferative response to mitogen of lymphocytes in patients with depression. However, when we analyzed if there were any correlation between stress with NCI and gender, we only found a significant association for women (Table 7). This might point out that women will have a worse neutrophil chemotaxis response, i.e., a reduced response against infection. On the other hand, the negative correlation found between depression and NK activity was found to be independent of patient gender.

One study limitation is that our patients are quite homogeneous regarding age (we have a variation coefficient of 19%), so we cannot separate them into different groups by this factor. However, we have found a negative correlation between age and NCI or PAN in our group of patients. This result agrees with the widely accepted fact that aging deteriorates immunological response (Khanfer et al., 2010; Fülöp et al., 2016). Another limitation of our study is that women are almost double than men. We recognize that this is not the ideal situation, and the gender groups should be more homogeneous to get stronger conclusions. Finally, we realize that the best way to analyze gender impact in our study is when there has been a regression model (moderation analysis) that would clarify whether or not gender is an independent factor that produces changes in stress, depression, and immunity. We could not perform this analysis because the only demographic variable is “age,” and we did not find any differences regarding this variable between men and women. However, it is also true that at least in our social context, more women come to our antiaging unit than men for undetermined reasons. Thus, our results might be also important for similar situations where more women than men were under evaluation.

## Conclusion

Our study confirms reported data, which demonstrated that stress and depression affect the nonspecific immune system underlining the alteration of NK cytotoxic response. Another goal of this intervention would be to preserve and improve physical and cognitive capacities, which would make that the biological age of the patient will be far from chronologic age and thus keep them away from disease. In this regard, interventions such as meditation exercise program should be tested as new tools to alleviate or prevent stress and depression effects. Careful monitoring of patient status will help to a better evaluation of the results of psychological programs regarding stress, depression, and immune system status. Thus, we could improve physical and psychological capacities, disease resistance, and improve patient life quality.

## Data Availability Statement

The raw data supporting the conclusions of this article will be made available by the authors, without undue reservation, to any qualified researcher.

## Ethics Statement

The studies involving human participants were reviewed and approved by the Scientific-Ethical Board of the center (Fundación Tejerina, Madrid) approved this study. The patients/participants provided their written informed consent to participate in this study.

## Author Contributions

BC-G recruited the patients, performed the experiments, analyzed the data, and wrote the manuscript. AF-N performed the statistical analysis. JR reviewed the manuscript. VM-F carried out the design and direction of the study and reviewed the statistical analysis and manuscript. All authors approved the final version of the manuscript.

## Conflict of Interest

The authors declare that the research was conducted in the absence of any commercial or financial relationships that could be construed as a potential conflict of interest.

## Abbreviations

C.p.m.: counts per minute
LCI: Lymphocyte Chemotaxis Index
LP: lymphocyte proliferation
NCI: Neutrophil Chemotaxis Index
NK: natural killer
NPI: Neutrophil Phagocytic Index
PAN: phagocytic activity of neutrophils
PHA: phytohemaglutinin
PNI: psychoneuroimmunology
PSQ: Perceived Stress Questionnaire.
